# Incorporation of Omega-3 Fatty Acids Into Human Skeletal Muscle Sarcolemmal and Mitochondrial Membranes Following 12 Weeks of Fish Oil Supplementation

**DOI:** 10.3389/fphys.2019.00348

**Published:** 2019-03-29

**Authors:** Christopher J. Gerling, Kazutaka Mukai, Adrian Chabowski, George J. F. Heigenhauser, Graham P. Holloway, Lawrence L. Spriet, Sebastian Jannas-Vela

**Affiliations:** ^1^Department of Human Health and Nutritional Sciences, University of Guelph, Guelph, ON, Canada; ^2^Department of Physiology, Medical University of Białystok, Białystok, Poland; ^3^Department of Medicine, McMaster University, Hamilton, ON, Canada; ^4^Exercise Science Laboratory, School of Kinesiology, Faculty of Medicine, Universidad Finis Terrae, Santiago, Chile

**Keywords:** omega-3, fatty acids, membrane, skeletal muscle, mitochondria, sarcolemma

## Abstract

Fish oil (FO) supplementation in humans results in the incorporation of omega-3 fatty acids (FAs) eicosapentaenoic acid (EPA; C20:5) and docosahexaenoic acid (DHA; C20:6) into skeletal muscle membranes. However, despite the importance of membrane composition in structure–function relationships, a paucity of information exists regarding how different muscle membranes/organelles respond to FO supplementation. Therefore, the purpose of the present study was to determine the effects 12 weeks of FO supplementation (3g EPA/2g DHA daily) on the phospholipid composition of sarcolemmal and mitochondrial fractions, as well as whole muscle responses, in healthy young males. FO supplementation increased the total phospholipid content in whole muscle (57%; *p* < 0.05) and the sarcolemma (38%; *p* = 0.05), but did not alter the content in mitochondria. The content of omega-3 FAs, EPA and DHA, were increased (+3-fold) in whole muscle, and mitochondrial membranes, and as a result the omega-6/omega-3 ratios were dramatically decreased (-3-fold), while conversely the unsaturation indexes were increased. Intriguingly, before supplementation the unsaturation index (UI) of sarcolemmal membranes was ∼3 times lower (*p* < 0.001) than either whole muscle or mitochondrial membranes. While supplementation also increased DHA within sarcolemmal membranes, EPA was not altered, and as a result the omega-6/omega-3 ratio and UI of these membranes were not altered. All together, these data revealed that mitochondrial and sarcolemmal membranes display unique phospholipid compositions and responses to FO supplementation.

## Introduction

Phospholipids are the major constituents of cell membranes (Singer and Nicolson, 1972), and their composition determines the structure and function of the cell (McIntosh and Simon, 2006). Changes in membrane composition affect permeability and fluidity of the membrane and influence interactions between membrane bound lipids and proteins, thereby impacting a myriad of physiological processes (Lee, 1998; Sprong et al., 2001; Andersen and Koeppe, 2007). The omega-3 fatty acids (FAs), eicosapentaenoic (EPA; C20:5) and docosahexaenoic (DHA; C20:6) acid, are characterized by their ability to modify the composition of biological membranes. Incorporation of these FAs has been demonstrated to modify the activity of enzymes and proteins involved in the transport of substrates and ions across membranes (Liu et al., 1994; Hulbert, 2007; Herbst et al., 2014; Shaikh et al., 2015; Chorner et al., 2016), and to disrupt the formation of membrane rafts, thereby modulating multiple cellular and signaling events in different tissues (Wassall and Stillwell, 2008; Williams et al., 2012).

Skeletal muscle is an essential tissue for the production of energy and to power contractions that provide the ability to move. Sarcolemmal and mitochondrial membranes represent key control points in both carbohydrate and FA metabolism. Sarcolemmal membranes are predominantly composed of saturated fatty acids (SFAs) and to a lesser extent of polyunsaturated fatty acids (PUFAs) (omega-6 > omega-3 FAs) and monounsaturated fatty acids (MUFAs) (Fiehn et al., 1971; Liu et al., 1994; Fajardo et al., 2013). Meanwhile mitochondrial membranes are composed evenly of SFAs and PUFAs, and the omega-6 FAs are the major PUFA constituent (Fiehn et al., 1971; Stefanyk et al., 2010; Fajardo et al., 2013; Herbst et al., 2014). These differences may reflect the diverse roles that these compartments play in the cell. Furthermore, there is substantial evidence revealing that EPA and DHA are significantly incorporated into skeletal muscle membranes following omega-3 FA supplementation, at the expense of omega-6 FAs (Liu et al., 1994; Andersson et al., 2002; Haugaard et al., 2006; Tsalouhidou et al., 2006; Smith G.I. et al., 2011; Herbst et al., 2014; McGlory et al., 2014; Chorner et al., 2016). However, most studies examining skeletal muscle sarcolemmal and mitochondrial membranes were done in rodent samples. Furthermore, it remains unknown if sarcolemmal and mitochondrial membranes similarly respond to omega-3 FA supplementation.

Therefore, the purpose of the present study was to determine phospholipid composition and the effects of fish oil (FO) on whole muscle, sarcolemmal and mitochondrial fractions in skeletal muscle from healthy young males. A portion of the sarcolemmal and mitochondrial data were published previously (Gerling et al., 2014; Herbst et al., 2014). We hypothesized that membrane composition would be different between membrane fractions, and that after FO supplementation, total phospholipid content would increase in the sarcolemma and that EPA and DHA content would be substantially augmented in whole muscle, sarcolemmal and mitochondrial membranes.

## Materials and Methods

This study was part of a larger project designed to address the effects of FO supplementation on whole body and skeletal muscle metabolism. Western blotting data from giant sarcolemmal vesicles and a portion of mitochondrial data were published previously (Gerling et al., 2014; Herbst et al., 2014). Specifically, the studies reported purity of mitochondria and giant sarcolemmal vesicle isolation by revealing presence or absence of known mitochondrial (oxidative phosphorylation proteins, E1α subunit pyruvate dehydrogenase), plasma membrane (caveolin-1) and sarcoplasmic reticulum (Ca^2+^ ATPase) proteins. In addition, Gerling et al. (2014) reported the effects of FO supplementation on whole muscle protein content of fatty acid translocase (FAT/CD36), plasma membrane fatty acid binding protein (FABPpm), and fatty acid transport proteins 1 and 4 (FATP1-4); sarcolemmal protein content of FAT/CD36, FABPpm, and FATP4; and mitochondrial content of FAT/CD36 and uncoupling protein 3. Lastly, Herbst et al. (2014) reported the effects of FO supplementation on absolute phospholipid composition of omega-6 and omega-3 FAs from isolated mitochondria.

### Subjects

Ten healthy, recreationally active males (age = 23.4 ± 1.4 year; body mass = 79.7 ± 3.8 kg; height = 180.4 ± 2.3 cm) volunteered to participate in the study. Written informed consent was received from each subject following a detailed explanation of the experimental protocol and any associated risks. Subjects were screened to ensure they were in good health, were not currently taking omega-3 supplements, had no previous history of omega-3 supplementation, and were not currently or had previously consumed a diet high in omega-3 FAs. Subjects were instructed to maintain consistent diet and exercise habits throughout the study. The study was approved by the University of Guelph and McMaster University Research Ethics Boards. All participants gave their informed consent prior to their inclusion in the study.

### Study Design

Two hours prior to arriving at the laboratory, participants were provided with a standardized meal consisting of a whole-wheat bagel with cream cheese and 500 ml of water. Subjects were asked to refrain from any physical activity, alcohol, and caffeine consumption 24 h prior to receiving muscle biopsies, and to consume a balanced diet [∼50% of energy (E) from carbohydrate, ∼30% E from fat, and ∼20% E from protein] the day before. Diet records were obtained from the day before pre-supplementation biopsies, and subjects were instructed to follow the same diet the day before post-supplementation biopsies. Following pre-supplementation biopsies, subjects consumed 5 capsules of Omega-3 Complete (1,000 mg per capsule, Jamieson Laboratories Ltd., Windsor, ON, Canada) per day for 12 weeks. Each capsule provided 400 mg of EPA and 200 mg of DHA in triglyceride (TG) form, for a total of 2,000 mg/days of EPA and 1,000 mg/days of DHA. Subjects were instructed to take 2 capsules in the morning with breakfast and 3 with evening dinner.

To promote supplement compliance, the participants were only given 2 weeks of capsules at a time. Written and oral reminders were also provided on a regular basis to ensure diet and exercise practices were maintained consistent throughout the study. Skeletal muscle biopsies were taken at baseline and following the 12-week supplementation period.

### Muscle Biopsies

Two to four resting muscle biopsies (total ∼500 mg) were obtained under local anesthesia (2% lidocaine without epinephrine) from the vastus lateralis muscle, using the percutaneous needle biopsy technique (Bergstrom, 1975). Pre-and post-supplementation biopsies were obtained from opposite legs. One muscle aliquot (∼200–250 mg) was used to isolate the sarcolemmal membrane by preparing giant sarcolemmal vesicles; a second aliquot (∼200 mg) was used for the isolation of mitochondria, and a third aliquot (∼100 mg) was immediately frozen for whole muscle analyses.

### Preparation of Giant Sarcolemmal Vesicles

Giant sarcolemmal vesicles were generated as described previously (Bonen et al., 2000; Talanian et al., 2010). Briefly, the tissue was cut into thin layers ∼1-3 mm thick and incubated for 1 h at 34°C in 140 mM KCl/10 mM MOPS (pH 7.4), 1 mL of collagenase (type VII, 150 units/ml), and aprotinin (30 μg/mL) in a shaking water bath. Following incubation, the supernatant fraction was collected. The remaining tissue was washed with KCl MOPS and 10 mM EDTA, resulting in a second supernatant fraction. The two supernatant fractions were pooled, and Percoll (G.E. Healthcare, Baie d’Urfé, QC, Canada), KCl, and aprotinin were added to final concentrations of 3.5% (w/v), 20 mM, and 10 μg/mL, respectively. The resulting suspension was placed at the bottom of a density gradient consisting of a 3 ml middle layer of 4% Nycodenz (v/v) and a 1 mL upper layer of KCl/MOPS. The sample was then centrifuged at 60 ×*g* for 45 min at room temperature. The vesicles were then harvested from the interface of the upper and middle solutions, diluted in KCl/MOPS, and re-centrifuged at 12,000 ×*g* for 5 min. The resultant vesicle pellet was re-suspended in KCl/MOPS and stored at -80°C for lipid analyses.

### Mitochondrial Isolation

Intact, pooled mitochondria [containing both intermyofibrillar (IMF) and subsarcolemmal (SS) fractions] were isolated as described previously (Holloway et al., 2007; Talanian et al., 2010). Briefly, fresh muscle was homogenized and centrifuged at 800 *g* for 10 min to separate SS and IMF fractions. The IMF mitochondrial fraction was treated with protease (Subtilisin A; Sigma, St. Louis, MO, United States) for exactly 5 min and centrifuged to remove the myofibrils. IMF and SS fractions were recombined, centrifuged twice at 10,000 *g* for 10 min and resuspended in 100 μl S&M solution (225 mm mannitol, 75 mm sucrose, 10 mm Tris–HCl, 0.1 mm EDTA; pH 7.4). Mitochondria were further purified using a percoll gradient and pooled for analysis.

### Lipid Analyses

Briefly, total lipids from the samples (whole muscle, GSV and isolated mitochondria fractions) were extracted (Folch et al., 1957), and thin-layer chromatography was used to separate individual classes of phospholipids (phosphatidylcholine, PC; phosphatidylethanolamine, PE; cardiolipin, CL; phosphatidylinositol, PI; phosphatidylserine, PS; and sphingomyelin, SM). Once isolated, phospholipids were methylated with 1 M methanolic sodium methoxide (Fluka) at room temperature for 10 min (Mahadevappa and Holub, 1987), and the fatty acid composition of each class was analyzed by gas chromatography (Hewlett-Packard 5890 Series II system, equipped with a double flame ionization detector and Agilent CP-Sil 88 capillary column, 100 m, internal diameter of 0.25 mm). Fatty acids were identified by comparison of retention times with those of a known standard, and absolute amounts of individual fatty acids were calculated with the aid of the internal standard, a pentadecanoic acid (Sigma–Aldrich, St. Louis, MO, United States) added to the samples before the methylation process by a single point quantification method. Total amounts of each class of the phospholipids were determined from the summed amounts of FAs in each phospholipid relative to protein concentration (nmol/mg protein) for sarcolemmal and mitochondrial preparations. Whole muscle amounts of phospholipid classes were determined relative to dry weight (nmol/g of dry mass). All fractions were also expressed as a percentage (%) of total FAs. The degree of unsaturation (unsaturation index, UI) of each muscle fraction was calculated as Σ*m*_i_ ×*n*_i_, where *m*_i_ is the mole percentage and *n*_i_ is the number of carbon–carbon double bonds of the FA.

### Statistical Analyses

All data are presented as means ± SEM and were checked for normality before any analyses. If data were normally distributed a paired two-tailed *t*-test was performed on each membrane fraction to determine the effects of FO supplementation on phospholipid composition. When data was not normally distributed a Wilcoxon matched-pair signed rank test was performed. A two-way ANOVA was used to compare the effects of FO supplementation on unsaturation index (UI) and omega-6/omega-3 ratio. When significance was found, Fisher’s LSD *post hoc* tests were used. Statistical significance was accepted at *p* < 0.05.

## Results

### Phospholipid Head Groups

**FIGURE 1:**
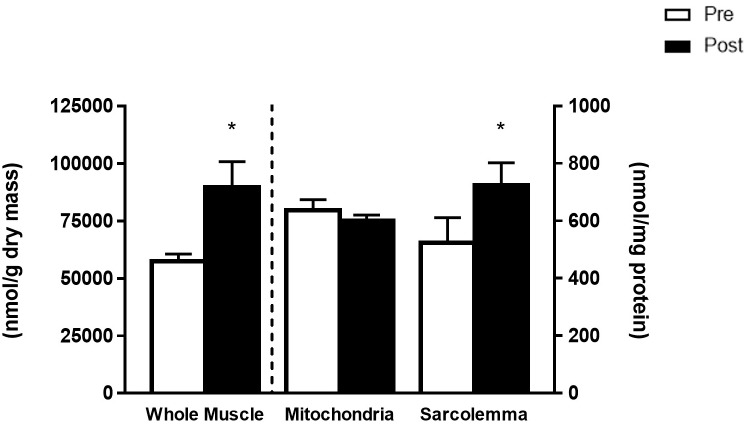
Total phospholipid species from whole muscle (left *y*-axis, nmol/g dry mass) and mitochondria and sarcolemma (right *y*-axis, nmol/mg protein) before (Pre) and after (Post) 12 weeks of fish oil supplementation. Values represent means ± SEM. ^∗^Significantly different from Pre.

The FO intervention increased the total phospholipid content of whole muscle membranes (56%) and sarcolemmal membranes (38%) (*p* < 0.05), while the mitochondrial membranes remained unaltered (Figure 1). The changes in total phospholipid head groups were due to increased abundance of all phospholipid head groups in whole muscle, and to increases in PI and CL in sarcolemma (Table 1).

**Table 1:** Total content and percent (%) distribution of phospholipid species from whole muscle, mitochondrial and sarcolemmal membrane fractions before (Pre) and after (Post) 12 weeks of fish oil supplementation.

	Whole muscle nmol/g dry mass		Mitochondria nmol/mg protein		Sarcolemma nmol/mg protein	
	Pre	Post	Pre	Post	Pre	Post
SM	1934 ± 97	2544 ± 254^∗^	22 ± 2	13 ± 1^∗^	45 ± 9	33 ± 3
PC	32014 ± 2104	45824 ± 5837^∗^	242 ± 16	243 ± 8	110 ± 16	121 ± 16
PS	2383 ± 136	3210 ± 282^∗^	33 ± 6	34 ± 5	139 ± 27	191 ± 36
PI	4738 ± 343	6567 ± 710	50 ± 4	49 ± 3	88 ± 18	172 ± 34^∗^
PE	11619 ± 705	18830 ± 1960^∗^	188 ± 19	179 ± 16	159 ± 33	207 ± 31
CL	5200 ± 1012	14270 ± 3122^∗^	102 ± 14	82 ± 6	38 ± 6	69 ± 11^∗^
	**Whole muscle (%)**		**Mitochondria (%)**		**Sarcolemma (%)**	
	**Pre**	**Post**	**Pre**	**Post**	**Pre**	**Post**

SM	3.5 ± 0.2	2.9 ± 0.1^∗^	3.6 ± 0.5	2.3 ± 0.2^∗^	6.8 ± 1.3	4.1 ± 0.6^∗^
PC	55.9 ± 1.7	51 ± 1.7^∗^	37.9 ± 0.9	40.6 ± 1.1	19.8 ± 3.3	15.1 ± 2.1
PS	4.2 ± 0.2	3.7 ± 0.2^∗^	5.6 ± 1.2	5.8 ± 0.9	25.6 ± 4.5	22.5 ± 4.5
PI	8.3 ± 0.4	7.4 ± 0.2^∗^	7.9 ± 0.4	8.3 ± 0.7	17.9 ± 2.4	23.2 ± 3.7^∗^
PE	20.3 ± 0.6	21.4 ± 1	29.2 ± 1.3	29.4 ± 1.7	23.7 ± 4.2	26.6 ± 5.1
CL	7.9 ± 1.6	13.5 ± 2.4^∗^	15.8 ± 2.1	13.6 ± 1	6.2 ± 1.2	8.5 ± 1.7^∗^


*Values represent means ± SEM. ^∗^Significantly different from Pre. SM, Sphingomyelin; PC, phosphatidylcholine; PS, phosphatidylserine; PI, phosphatidylinositol; PE, phosphatidylethanolamine; CL, cardiolipin.*

When expressed as a percent of total FAs, phospholipid species from whole muscle membranes from most prevalent to least were PC (56%), PE (20%), CL (8%), PI (8%), PS (4%), and SM (4%) (Table 1). After FO supplementation, there was a decrease in percent fraction of SM, PC, PS and PI, and an increase in CL (Table 1). In mitochondria the distribution of phospholipids from highest to lowest were PC (38%), PE (29%), CL (16%), PI (8%), PS (6%), and SM (3%). There was no effect of FO supplementation in phospholipid head group distribution of mitochondria, except for SM, which was decreased (*p* < 0.05; Table 1). Lipid head groups from sarcolemmal membranes were more uniform, as PS, PE, PC and PI had similar percent fractions (18–25%), followed by SM (7%) and CL (6%). After FO supplementation, there was a decrease in the percent fraction of SM, and an increase in the distribution of PI and CL (*p* < 0.05; Table 1).

### Absolute and Relative Content of Fatty Acids

**FIGURE 2:**
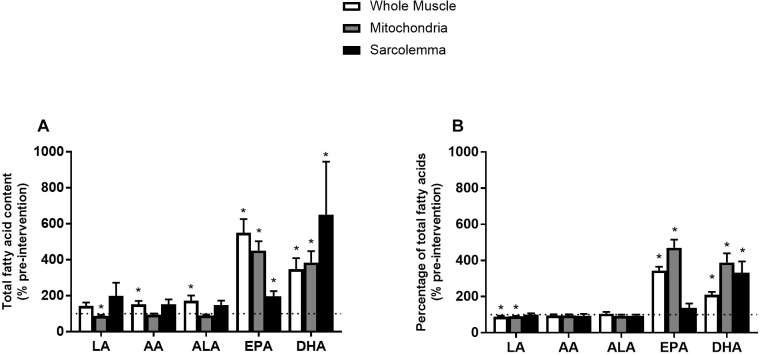
Percent (%) of absolute total fatty acid content **(A)** and % of pre-intervention relative total fatty acid content **(B)** from whole muscle, sarcolemma and mitochondria membrane fractions after 12 week fish oil supplementation. LA, Linoleic acid; AA, arachidonic acid; ALA, alpha-linolenic acid; EPA, eicosapentaenoic acid; DHA, docosahexaenoic acid. Values represent means ± SEM. ^∗^Significantly different from pre-supplementation (100%).

Most studies analyzing skeletal muscle membrane composition have expressed individual fatty acyl tails as a % of total FAs (relative) opposed to a concentration basis (absolute). However, there is evidence that membrane composition differs when lipids are expressed in relative or absolute terms (Schwertner and Mosser, 1993). Therefore, we sought to analyze individual FAs in both absolute and relative terms. Phospholipids from whole muscle membranes were predominantly composed of omega-6 FAs and SFA, followed by MUFA and omega-3 FAs (Table 2). After FO supplementation, there was an increase in absolute abundance of SFA, MUFA and omega-3 FAs. The increases in omega-3 FAs were attributed to elevations in EPA and DHA (Figure 2). When expressed as a percent of total FAs, SFA and omega-6 FAs were decreased after supplementation, while omega-3 FAs remained elevated. The relative changes in omega-6 and omega-3 FAs were attributed to linoleic acid (LA), and EPA and DHA, respectively (Figure 2B and Table 2).

**Table 2:** Total content and percent (%) distribution of fatty acyl tails from whole muscle, mitochondrial and sarcolemmal membrane fractions before (Pre) and after (Post) 12 weeks of fish oil supplementation.

	Whole muscle nmol/g dry mass		Mitoch=ondria nmol/mg protein		Sarcolemma nmol/mg protein	
	Pre	Post	Pre	Post	Pre	Post
SFA	24450 ± 1348	35852 ± 4416^∗^	291 ± 11	272 ± 10^∗^	395 ± 65	562 ± 65^∗^
MUFA	4522 ± 337	9033 ± 1576^∗^	38 ± 2	34 ± 1^∗^	48 ± 9	61 ± 6
n-6	26442 ± 1764	37350 ± 4742	293 ± 31	249 ± 19	67 ± 14	78 ± 9
n-3	1895 ± 117	7424 ± 1007^∗^	14 ± 2	45 ± 5^∗^	15 ± 3	23 ± 3^∗^
	**Whole muscle (%)**		**Mitochondria (%)**		**Sarcolemma (%)**	
	**Pre**	**Post**	**Pre**	**Post**	**Pre**	**Post**

SFA	42.8 ± 0.9	40.0 ± 1.0^∗^	46.6 ± 2.5	45.7 ± 2.1	76.1 ± 2.0	76.9 ± 1.9
MUFA	7.9 ± 0.4	10.1 ± 1.1	6.1 ± 0.4	5.7 ± 0.4	9.0 ± 0.6	8.8 ± 0.8
n-6	46.0 ± 1.1	41.6 ± 0.8^∗^	45.1 ± 2.8	41.1 ± 2.6^∗^	11.9 ± 1.3	11.0 ± 0.9
n-3	3.3 ± 0.2	8.3 ± 0.6^∗^	2.2 ± 0.2	7.5 ± 0.7^∗^	3.0 ± 0.4	3.3 ± 0.4


*Values represent means ± SEM. ^∗^Significantly different from Pre. SFA, Saturated fatty acids; MUFA, Monounsaturated fatty acids; n-6, omega-6; n-3, omega-3*.

Similar to whole muscle, mitochondrial membranes were mostly composed of SFA and omega-6 FAs, and to a lesser extent of MUFA and omega-3 FAs (Table 2). After the FO intervention, there was an absolute increase in total content of omega-3 FAs (EPA and DHA) alongside a decrease in SFA and MUFAs (Figure 2A and Table 2). When expressed in relative terms, EPA and DHA remained elevated, while omega-6 FAs were decreased, mainly due to a reduction in LA. Interestingly, FAs in PI and CL were resistant to change, as only PI increased. Therefore, alterations in the content of omega-3 fatty acyl tails from mitochondrial membranes were attributed to PC, PS, and PE (Supplementary Tables).

Sarcolemmal membranes were primarily composed of SFA followed by omega-6 FAs, MUFA and omega-3 FAs (Table 2). After FO treatment, the absolute contents of SFA, EPA, DHA and total omega-3 FAs were increased (*p* < 0.05). However, when expressed in relative terms only DHA was increased (*p* < 0.05; Figure 2 and Table 2).

### Omega-6/Omega-3 Ratio and Unsaturation Index

**FIGURE 3:**
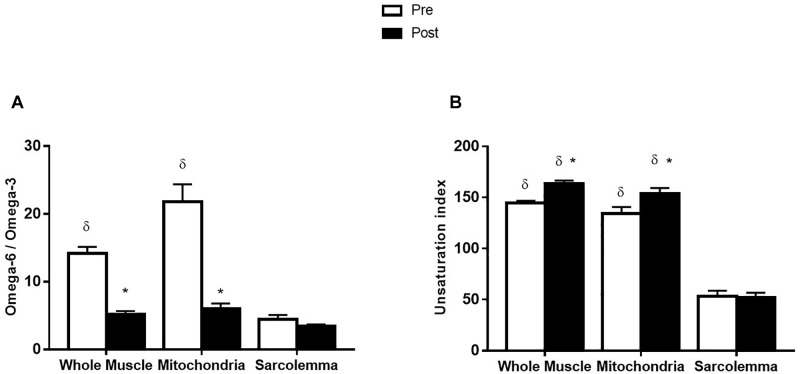
Omega-6/omega-3 ratio **(A)** and unsaturation index **(B)** from whole muscle, sarcolemma and mitochondria membrane fractions before (Pre) and after (Post) 12 week fish oil supplementation. Values represent means ± SEM. ^∗^Significantly different from Pre. ^δ^Significantly different to sarcolemma.

Before supplementation, the ratio of omega-6/omega-3 from whole muscle was lower than mitochondria (*p* < 0.001). Additionally, the omega-6/omega-3 ratio was lower in the sarcolemma compared to whole muscle and mitochondria (*p* < 0.001; Figure 3A). After the supplementation period, the omega-6/omega-3 ratio in whole muscle and mitochondrial membrane fractions were decreased (*p* < 0.001) and similar to the omega-6/omega-3 ratio of sarcolemma, which was not affected by supplementation (Figure 3A). The UI from sarcolemma, was ∼3-times lower than whole muscle and mitochondria (*p* < 0.001; Figure 3B). After omega-3 supplementation UI was not affected in sarcolemma; however, UI was increased (14%; *p* < 0.01) in whole muscle and mitochondrial membranes (Figure 3B).

## Discussion

The main findings of the present study were that: (1) a 12-week FO supplementation period increased the total phospholipid content in whole muscle and sarcolemma, but did not affect the mitochondrial fraction, (2) omega-3 FAs were substantially increased in whole muscle, mitochondrial and sarcolemmal membrane fractions, and (3) sarcolemmal membranes had a lower UI and the FA composition was less responsive to omega-3 supplementation than whole muscle and mitochondria.

### Membrane Phospholipid Compositions in Skeletal Muscle

In the present study, whole muscle and mitochondrial membranes had a similar composition, as ∼85% of phospholipids were distributed between PC, PE, and CL, and over 80% of phospholipid fatty acyl tails were composed of SFA and omega-6 FAs. These results support previous findings in mitochondria (Tsalouhidou et al., 2006; Stefanyk et al., 2010; Holloway et al., 2012) and whole muscle (Andersson et al., 2002; Smith G.I. et al., 2011; McGlory et al., 2014) from rodent and human tissue. Meanwhile, sarcolemmal membranes were predominantly composed of PC (20%), PS (26%), PI (18%), and PE (24%), and to a lesser extent of CL and SM (<10% each). Approximately 76% of individual phospholipid fatty acyl tails from sarcolemmal vesicles were comprised of SFAs, followed by omega-6 FAs (12%), MUFA (9%), and omega-3 FAs (3%). These results are similar to a recent study that isolated plasma membrane from rodent tissue by mechanically skinning muscle fibers (Fajardo et al., 2013). However, there were major differences regarding content of SM and MUFAs, as they were ∼3 times higher in their study. These differences could be due to the host used (human vs. rodent), muscle fiber type analyzed (mixed vs. type II), region of sarcolemma isolated and isolation procedure.

### Effect of Omega-3 FAs on Skeletal Muscle Phospholipids

In the present study, 12 weeks of FO supplementation increased the total phospholipid content of whole muscle and sarcolemma, but not mitochondrial membranes. Specifically, within whole muscle all phospholipid head groups were increased, propelled by increases in SFA, MUFAs and omega-3 FAs. While in sarcolemma, PI and CL were increased, driven by increases in SFA and omega-3 FAs. Although the mechanisms behind these changes are currently unknown, omega-3 FAs have been demonstrated to increase expression of several proteins involved in diverse metabolic processes that have been linked to skeletal muscle anabolism, inflammation, and carbohydrate and lipid metabolism (Deckelbaum et al., 2006; Jeromson et al., 2015). Although speculative, omega-3 FAs may have increased the expression of proteins involved in phospholipid synthesis, augmenting the content of whole muscle and sarcolemmal fractions. While it remains unknown why mitochondrial membranes were not altered, importantly previous research has demonstrated that omega-3 supplementation does not affect mitochondrial content/biogenesis (Herbst et al., 2014; Matravadia et al., 2014; Johnson et al., 2015), supporting the current data.

Omega-3 supplementation is well-known to alter the lipid composition of skeletal muscle membrane fractions (Liu et al., 1994; Andersson et al., 2002; Haugaard et al., 2006; Tsalouhidou et al., 2006; Smith G.I. et al., 2011; Herbst et al., 2014; McGlory et al., 2014; Chorner et al., 2016). In the present study we aimed to determine if sarcolemmal and mitochondrial membranes were similarly altered with FO supplementation. While omega-3 FAs were robustly increased in all three membrane fractions when expressed in absolute concentrations, when expressed in relative terms, EPA and DHA remained elevated in whole muscle and mitochondrial fractions, and only DHA in the sarcolemmal fraction. While it has been suggested that expressing phospholipids in absolute terms removes the influence from other FAs, improving accuracy for interpreting changes in phospholipid composition (Schwertner and Mosser, 1993), this approach prevents comparisons between membrane fractions which are normalized differently (e.g., dry weight vs. mg protein). Therefore, the relative changes observed strongly suggest that fatty acid composition of sarcolemmal membranes is less affected by FO supplementation, an interpretation further supported by the absence of a change in the UI. In contrast the omega-6/omega-3 ratio decreased and UI increased with FO supplementation in the whole muscle and mitochondrial fractions, similar to previous studies (Andersson et al., 2002; Dangardt et al., 2012; Chorner et al., 2016). All together these data suggest that sarcolemmal membranes are more resistant to change, most probably due to the low omega-6/omega-3 ratio prior to supplementation. This is further reinforced as the ratio appeared similar across all membrane fractions after FO supplementation.

The presence of CL in sarcolemmal vesicles was surprising as it is believed to be only expressed in mitochondrial membranes (Post et al., 1995). A potential explanation could be due to minor contamination from other membrane fractions, as the presence of mitochondrial and sarcoplasmic reticulum proteins using this isolation procedure has been previously shown (Smith B.K. et al., 2011). In the current study, sarcolemmal and mitochondrial membrane compositions were different and responded differently to omega-3 supplementation, suggesting that the differences between these compartments were not exclusively a result of contamination from other fractions and likely resulted in an underestimation of the differences between compartments. Another potential explanation is that this preparation may isolate a specific domain of the sarcolemma, probably caveolae or lipid rafts, were CL may be required for proper assembly. We and others have observed significant presence of lipids (SFAs and SM) and proteins (caveolin-1, FAT/CD36 and glucose transporter 4) known to be expressed in lipid rafts and caveolae (Ploug et al., 1993; Song et al., 1996; Koonen et al., 2002; Vistisen et al., 2004; Pike, 2006; Glatz et al., 2010; Briolay et al., 2013) within the sarcolemmal preparation. Furthermore, it has been observed that CL can relocate to the plasma membrane during periods of apoptosis or mitophagy (Sorice et al., 2004; Schlame and Greenberg, 2017). In skeletal muscle a significant portion (∼20%) of mitochondria reside close to the sarcolemma. Therefore, it is possible that contact sites within sarcolemmal and mitochondrial membranes instigate transport and relocation of CL to specific domains in sarcolemma. Clearly research delineating the mechanistic aspects of CL within sarcolemmal membranes is required to advance our understanding for the role of lipid species in metabolic homeostasis.

The biological significance of membrane changes after omega-3 supplementation is complex and not completely understood. Analysis of structural and compositional properties of membranes has shown that these properties can affect important metabolic and physiological processes such as protein activity, ion homeostasis, carrier-mediated transport, signal transduction, and membrane assembly (Lee, 1998; McIntosh and Simon, 2006; Williams et al., 2012). It is likely that incorporation of omega-3 FAs into skeletal muscle membranes may result in changes in localization and/or post-translational modifications of membrane proteins, altering divergent metabolic processes, including sarcolemmal substrate transport (Chorner et al., 2016; Jeromson et al., 2017), mitochondrial function (Herbst et al., 2014) and protein synthesis (McGlory et al., 2019). Future research examining the physiological effects of the omega-based compositional changes in the various cellular compartments that occur with supplementation in human skeletal muscle is warranted.

## Conclusion

In conclusion, this study found that a 12-week FO supplementation period increased the total phospholipid content in whole muscle and sarcolemma but did not affect the mitochondrial fraction. The omega-3 fatty acids, EPA and DHA, increased in whole muscle, mitochondrial and sarcolemmal membrane fractions. Sarcolemmal membranes appeared to be less responsive than whole muscle and mitochondria, likely due to the low omega-6/omega-3 ratio. These data implicate the importance of studying individual membranes independent of whole muscle, as each membrane fraction revealed unique phospholipid composition, and responded in a different manner to FO supplementation.

## Data Availability

The datasets generated for this study are available on request to the corresponding author.

## Author Contributions

CG, GH, and LS contributed to the conception of the study. CG, KM, AC, GJH, GH, LS, and SJ-V assisted with measurements. CG, GH, LS, and SJ-V analyzed and interpreted the data, and wrote the first draft of the manuscript. All authors read and approved the final manuscript.

## Conflict of Interest Statement

The authors declare that the research was conducted in the absence of any commercial or financial relationships that could be construed as a potential conflict of interest.

## References

[B1] Andersen O. S. Koeppe R. E. (2007). Bilayer thickness and membrane protein function: an energetic perspective. *Annu. Rev. Biophys. Biomol. Struct.* 36 107–130. 10.1146/annurev.biophys.36.040306.132643 17263662

[B2] Andersson A. Nälsén C. Tengblad S. Vessby B. (2002). Fatty acid composition of skeletal muscle reflects dietary fat composition in humans. *Am. J. Clin. Nutr.* 76 1222–1229. 10.1093/ajcn/76.6.1222 12450886

[B3] Bergstrom J. (1975). Percutaneous needle biopsy of skeletal muscle in physiological and clinical research. *Scand. J. Clin. Lab. Invest.* 35 609–616. 10.3109/00365517509095787 1108172

[B4] Bonen A. Luiken J. J. Arumugam Y. Glatz J. F. Tandon N. N. (2000). Acute regulation of fatty acid uptake involves the cellular redistribution of fatty acid translocase. *J. Biol. Chem.* 275 14501–14508. 10.1074/jbc.275.19.14501 10799533

[B5] Briolay A. Jaafar R. Nemoz G. Bessueille L. (2013). Myogenic differentiation and lipid-raft composition of L6 skeletal muscle cells are modulated by PUFAs. *Biochim. Biophys. Acta Biomembr.* 1828 602–613. 10.1016/j.bbamem.2012.10.006 23079583

[B6] Chorner Z. Barbeau P. A. Castellani L. Wright D. C. Chabowski A. Holloway G. P. (2016). Dietary α-linolenic acid supplementation alters skeletal muscle plasma membrane lipid composition, sarcolemmal FAT/CD36 abundance, and palmitate transport rates. *Am. J. Physiol.* 11 R1234–R1242. 10.1152/ajpregu.00346.2016 27806984 PMC5256967

[B7] Dangardt F. Chen Y. Gronowitz E. Dahlgren J. Friberg P. Strandvik B. (2012). High physiological omega-3 fatty acid supplementation affects muscle fatty acid composition and glucose and insulin homeostasis in obese adolescents. *J. Nutr. Metab.* 2012:395757. 10.1155/2012/395757 22523671 PMC3317167

[B8] Deckelbaum R. J. Worgall T. S. Seo T. (2006). N-3 fatty acids and gene expression. *Am. J. Clin. Nutr.* 83 1520S–1525S. 10.3945/ajcn.110.001065.1874S 16841862

[B9] Fajardo V. A. McMeekin L. Basic A. Lamb G. D. Murphy R. M. LeBlanc P. J. (2013). Isolation of sarcolemmal plasma membranes by mechanically skinning rat skeletal muscle fibers for phospholipid analysis. *Lipids* 48 421–430. 10.1007/s11745-013-3770-x 23430510

[B10] Fiehn W. Peter J. B. Mead J. F. Gan-Elepano M. (1971). Lipids and fatty acids of sarcolemma, sarcoplasmic reticulum, and mitochondria from rat skeletal muscle. *J. Biol. Chem.* 246 5617–5620. 5096085

[B11] Folch J. Lees M. Sloane-Stanley G. H. (1957). A simple method for the isolation and purification of total lipids from animal tissues. *J. Biol. Chem.* 226 497–509. 13428781

[B12] Gerling C. J. Whitfield J. Mukai K. Spriet L. L. (2014). Variable effects of 12 weeks of omega-3 supplementation on resting skeletal muscle metabolism. *Appl. Physiol. Nutr. Metab.* 39 1083–1091. 10.1139/apnm-2014-0049 25054452

[B13] Glatz J. F. C. Luiken J. J. F. P. Bonen A. (2010). Membrane fatty acid transporters as regulators of lipid metabolism: implications for metabolic disease. *Physiol. Rev.* 90 367–417. 10.1152/physrev.00003.2009 20086080

[B14] Haugaard S. B. Madsbad S. Høy C. E. Vaag A. (2006). Dietary intervention increases n-3 long-chain polyunsaturated fatty acids in skeletal muscle membrane phospholipids of obese subjects. implications for insulin sensitivity. *Clin. Endocrinol.* 64 169–178. 10.1111/j.1365-2265.2006.02444.x 16430716

[B15] Herbst E. A. F. Paglialunga S. Gerling C. Whitfield J. Mukai K. Chabowski A. (2014). Omega-3 supplementation alters mitochondrial membrane composition and respiration kinetics in human skeletal muscle. *J. Physiol.* 592 1341–1352. 10.1113/jphysiol.2013.267336 24396061 PMC3961091

[B16] Holloway G. P. Fajardo V. A. McMeekin L. LeBlanc P. J. (2012). Unsaturation of mitochondrial membrane lipids is related to palmitate oxidation in subsarcolemmal and intermyofibrillar mitochondria. *J. Membr. Biol.* 245 165–176. 10.1007/s00232-012-9426-6 22527602

[B17] Holloway G. P. Thrush A. B. Heigenhauser G. J. F. Tandon N. N. Dyck D. J. Bonen A. (2007). Skeletal muscle mitochondrial FAT/CD36 content and palmitate oxidation are not decreased in obese women. *Am. J. Physiol. Endocrinol. Metab.* 292 E1782–E1789. 10.1152/ajpendo.00639.2006 17311893

[B18] Hulbert A. J. (2007). Membrane fatty acids as pacemakers of animal metabolism. *Lipids* 42 811–819. 10.1007/s11745-007-3058-0 17464520

[B19] Jeromson S. Gallagher I. J. Galloway S. D. R. Hamilton D. L. (2015). Omega-3 fatty acids and skeletal muscle health. *Mar. Drugs* 13 6977–7004. 10.3390/md13116977 26610527 PMC4663562

[B20] Jeromson S. Mackenzie I. Doherty M. K. Whitfield P. D. Bell G. Dick J. (2017). Lipid remodelling and an altered membrane proteome may drive the effects of EPA and DHA treatment on skeletal muscle glucose uptake and protein accretion. *Am. J. Physiol. Endocrinol. Metab.* 314 E605–E619. 10.1152/ajpendo.00438.2015 28655718

[B21] Johnson M. L. Lalia A. Z. Dasari S. Pallauf M. Fitch M. Hellerstein M. K. (2015). Eicosapentaenoic acid but not docosahexaenoic acid restores skeletal muscle mitochondrial oxidative capacity in old mice. *Aging Cell* 14 734–743. 10.1111/acel.12352 26010060 PMC4568961

[B22] Koonen D. P. Y. Coumans W. A. Arumugam Y. Bonen A. Glatz J. F. C. Luiken J. J. F. P. (2002). Giant membrane vesicles as a model to study cellular substrate uptake dissected from metabolism. *Mol. Cell. Biochem.* 239 121–130. 10.1023/A:1020549311455 12479577

[B23] Lee A. G. (1998). How lipids interact with an intrinsic membrane protein: the case of the calcium pump. *Biochim. Biophys. Acta* 1376 381–390. 10.1016/S0304-4157(98)00010-0 9804995

[B24] Liu S. Baracos V. E. Quinney H. A. Clandinin M. T. (1994). Dietary omega-3 and polyunsaturated fatty acids modify fatty acyl composition and insulin binding in skeletal-muscle sarcolemma. *Biochem. J.* 299 831–837. 10.1042/bj2990831 8192673 PMC1138096

[B25] Mahadevappa V. G. Holub B. J. (1987). Quantitative loss of individual eicosapentaenoyl-relative to arachidonoyl-containing phospholipids in thrombin-stimulated human platelets. *J. Lipid Res.* 28 1275–1280. 2828497

[B26] Matravadia S. Herbst E. A. F. Jain S. S. Mutch D. M. Holloway G. P. (2014). Both linoleic and α-linolenic acid prevent insulin resistance but have divergent impacts on skeletal muscle mitochondrial bioenergetics in obese Zucker rats. *Am. J. Physiol. Endocrinol. Metab.* 307 E102–E114. 10.1152/ajpendo.00032.2014 24844257

[B27] McGlory C. Galloway S. D. R. Hamilton D. L. McClintock C. Breen L. Dick J. R. (2014). Temporal changes in human skeletal muscle and blood lipid composition with fish oil supplementation. *Prostaglandins Leukot*. *Essent. Fat. Acids* 90 199–206. 10.1016/j.plefa.2014.03.001 24726616

[B28] McGlory C. Gorissen S. H. M. Kamal M. Bahniwal R. Hector A. J. Baker S. K. (2019). Omega-3 fatty acid supplementation attenuates skeletal muscle disuse atrophy during two weeks of unilateral leg immobilization in healthy young women. *FASEB J.* 33 4586–4597. 10.1096/fj.201801857RRR 30629458

[B29] McIntosh T. J. Simon S. A. (2006). Roles of bilayer material properties in function and distribution of membrane proteins. *Annu. Rev. Biophys. Biomol. Struct.* 35 177–198. 10.1146/annurev.biophys.35.040405.102022 16689633

[B30] Pike L. J. (2006). Rafts defined: a report on the Keystone symposium on lipid rafts and cell function. *J. Lipid Res.* 47 1597–1598. 10.1194/jlr.E600002-JLR200 16645198

[B31] Ploug T. Wojtaszewski J. Kristiansen S. Hespel P. Galbo H. Richter E. A. (1993). Glucose transport and transporters in muscle giant vesicles: differential effects of insulin and contractions. *Am. J. Physiol.* 264 E270–E278. 10.1152/ajpendo.1993.264.2.E270 8447394

[B32] Post J. A. Verkleij A. J. Langer G. A. (1995). Organization and function of sarcolemmal phospholipids in control and ischemic/reperfused cardiomyocytes. *J. Mol. Cell. Cardiol.* 27 749–760. 10.1016/0022-2828(95)90080-2 7776380

[B33] Schlame M. Greenberg M. L. (2017). Biosynthesis, remodeling and turnover of mitochondrial cardiolipin. *Biochim. Biophys. Acta Mol. Cell Biol. Lipids* 1862 3–7. 10.1016/j.bbalip.2016.08.010 27556952 PMC5125896

[B34] Schwertner H. A. Mosser E. L. (1993). Comparison of lipid fatty acids on a concentration basis vs weight percentage basis in patients with and without coronary artery disease or diabetes. *Clin. Chem.* 39 659–663. 8472362

[B35] Shaikh S. R. Wassall S. R. Brown D. A. Kosaraju R. (2015). N-3 polyunsaturated fatty acids, lipid microclusters, and vitamin E. *Curr. Top. Membr.* 75 209–231. 10.1016/bs.ctm.2015.03.003 26015284

[B36] Singer S. J. Nicolson G. L. (1972). The fluid mosaic model of the structure of cell membranes. *Science* 175 720–731. 10.1126/science.175.4023.720 4333397

[B37] Smith B. K. Jain S. S. Rimbaud S. Dam A. Quadrilatero J. Ventura-Clapier R. (2011). FAT/CD36 is located on the outer mitochondrial membrane, upstream of long-chain acyl-CoA synthetase, and regulates palmitate oxidation. *Biochem. J.* 437 125–134. 10.1042/BJ20101861 21463259

[B38] Smith G. I. Atherton P. Reeds D. N. Mohammed B. S. Rankin D. Rennie M. J. (2011). Dietary omega-3 fatty acid supplementation increases the rate of muscle protein synthesis in older adults: a randomized controlled trial. *Am. J. Clin. Nutr.* 93 402–412. 10.3945/ajcn.110.005611 21159787 PMC3021432

[B39] Song K. S. Scherer P. E. Tang Z. Okamoto T. Li S. Chafel M. (1996). Expression of caveolin-3 in skeletal, cardiac, and smooth muscle cells. *J. Biol. Chem.* 271 15160–15165. 10.1074/jbc.271.25.15160 8663016

[B40] Sorice M. Circella A. Cristea I. M. Garofalo T. Di Renzo L. Alessandri C. (2004). Cardiolipin and its metabolites move from mitochondria to other cellular membranes during death receptor-mediated apoptosis. *Cell Death Differ.* 11 1133–1145. 10.1038/sj.cdd.4401457 15181455

[B41] Sprong H. van der Sluijs P. van Meer G. (2001). How proteins move lipids and lipids move proteins. *Nat. Rev. Mol. Cell Biol.* 2 504–513. 10.1038/35080071 11433364

[B42] Stefanyk L. E. Coverdale N. Roy B. D. Peters S. J. Leblanc P. J. (2010). Skeletal muscle type comparison of subsarcolemmal mitochondrial membrane phospholipid fatty acid composition in rat. *J. Membr. Biol.* 234 207–215. 10.1007/s00232-010-9247-4 20336283

[B43] Talanian J. L. Holloway G. P. Snook L. A. Heigenhauser G. J. F. Bonen A. Spriet L. L. (2010). Exercise training increases sarcolemmal and mitochondrial fatty acid transport proteins in human skeletal muscle. *Am. J. Physiol. Endocrinol. Metab.* 299 E180–E188. 10.1152/ajpendo.00073.2010 20484014

[B44] Tsalouhidou S. Argyrou C. Theofilidis G. Karaoglanidis D. Orfanidou E. Nikolaidis M. G. (2006). Mitochondrial phospholipids of rat skeletal muscle are less polyunsaturated than whole tissue phospholipids: Implications for protection against oxidative stress. *J. Anim. Sci.* 84 2818–2825. 10.2527/jas.2006-031 16971584

[B45] Vistisen B. Roepstorff K. Roepstorff C. Bonen A. van Deurs B. Kiens B. (2004). Sarcolemmal FAT/CD36 in human skeletal muscle colocalizes with caveolin-3 and is more abundant in type 1 than in type 2 fibers. *J. Lipid Res.* 45 603–609. 10.1194/jlr.M300424-JLR200 14729862

[B46] Wassall S. R. Stillwell W. (2008). Docosahexaenoic acid domains: the ultimate non-raft membrane domain. *Chem. Phys. Lipids* 153 57–63. 10.1016/j.chemphyslip.2008.02.010 18343224

[B47] Williams J. A. Batten S. E. Harris M. Rockett B. D. Shaikh S. R. Stillwell W. (2012). Docosahexaenoic and eicosapentaenoic acids segregate differently between raft and nonraft domains. *Biophys. J.* 103 228–237. 10.1016/j.bpj.2012.06.016 22853900 PMC3400777

